# The occurrence and risk factors associated with post‐traumatic stress disorder among discharged COVID‐19 patients in Tianjin, China

**DOI:** 10.1002/brb3.2492

**Published:** 2022-01-22

**Authors:** Zaoxian Mei, Xiaohui Wu, Xueli Zhang, Xingjie Zheng, Wenxin Li, Rui Fan, Hongwei Yu, Shunming Zhang, Yeqing Gu, Xuena Wang, Yang Xia, Ge Meng, Jun Shen, Kaijun Niu

**Affiliations:** ^1^ Department of Tuberculosis Tianjin Haihe Hospital, Tianjin Institute of Respiratory Diseases No.890 JinGu Road, Jinnan District Tianjin 300350 China; ^2^ Nutritional Epidemiology Institute and School of Public Health Tianjin Medical University No.22 Qixiangtai Road, Heping District Tianjin 300070 China; ^3^ Tianjin Key Laboratory on Technologies Enabling Development of Clinical Therapeutics and Diagnostics College of Pharmacy Tianjin Medical University No.22 Qixiangtai Road, Heping District Tianjin 300070 China

**Keywords:** COVID‐19, post‐traumatic stress disorder

## Abstract

**Background:**

Post‐traumatic stress disorder (PTSD) is a serious mental health condition that is triggered by a terrifying event. We aimed to investigate the occurrence and risk factors of PTSD among discharged COVID‐19 patients.

**Methods:**

This study included 144 discharged COVID‐19 patients. PTSD was assessed by using validated cut‐offs of the impact of event scale‐revised (IES‐R, score ≥25). All patients completed a detailed questionnaire survey, and clinical parameters were routinely measured in the hospital. Binary logistic regression models were applied to identify factors associated with PTSD.

**Results:**

Of the 144 participants with laboratory‐confirmed COVID‐19, the occurrence of PTSD was 16.0%. In multivariable analyses, age above 40 years (adjusted OR [95% CI], 5.19 [2.17–12.32]), female sex (adjusted OR [95% CI], 7.82 [3.18–18.21]), current smoker (adjusted OR [95% CI], 6.72 [3.23–15.26]), and ≥3 involved pulmonary lobes (adjusted OR [95% CI], 5.76 [1.19–15.71]) were significantly associated with a higher risk of PTSD. Conversely, history of hypertension and serum hemoglobin levels were significantly associated with a lower risk of PTSD with adjusted ORs (95% CI) of 0.37 (0.12–0.87) and 0.91 (0.82–0.96), respectively.

**Conclusion:**

Old age, gender (being female), current smoking, bacterial pneumonia, and ≥3 involved pulmonary lobes were associated with an increased occurrence of PTSD among discharged COVID‐19 patients.

## Abstract 2

Post‐traumatic stress disorder (PTSD) is a serious mental health condition that is triggered by a terrifying event. This study showed that the occurrence of PTSD among patients with confirmed COVID‐19 infection was not rare. We found that several factors including age, gender (being female), current smoking, bacterial pneumonia, and ≥3 involved pulmonary lobes were associated with an increased occurrence of PTSD.

## 1 INTRODUCTION

Coronavirus disease 2019 (COVID‐19), caused by severe acute respiratory syndrome coronavirus 2 (SARS‐CoV‐2), has affected more than 213 countries worldwide, resulting in an ongoing global pandemic. According to the statistics available in Worldometer (Worldometer, 2020), as of July 20, 2020, COVID‐19 had caused more than 14 million cases and nearly 610,000 deaths in the world.

The COVID‐19 not only affects physical health but also causes mental health problems (Talevi et al., 2020; Vindegaard & Benros, 2020). One of the common psychological problems is post‐traumatic stress disorder (PTSD) arising from exposure to trauma (Pfefferbaum & North, 2020). Bo HX *et al*. reported that 96.2% out of 714 hospitalized stabile patients suffered from significant posttraumatic stress symptoms associated with the COVID‐19 before discharge (Bo et al., 2020). A previous study in Iraq and Afghanistan found that veterans who reported PTSD have greater difficulties in their romantic relationships, less family cohesion, less social support, poorer social functioning, and lower life satisfaction compared to subjects without PTSD (Tsai et al., 2012). Furthermore, PTSD was associated with attempted suicide and resulted in a high economic burden for society (Galea et al., 2005). Therefore, identifying risk factors of PTSD symptoms in confirmed COVID‐19 patients is important for the early detection of at‐risk subgroups and potential interventions.

Although several recent studies have investigated the factors associated with PTSD in front‐line medical staff (Wang et al., 2020; Wu & Wei, 2020), the general population (Wang et al., 2020), or university students (Tang et al., 2020), few studies have focused on the factors associated with PTSD among confirmed COVID‐19 patients. Thus, the related factors of the occurrence of PTSD in patients with confirmed COVID‐19 infection are unknown. To our knowledge, only one small cross‐sectional study (*n* = 41) on PTSD in COVID‐19 confirmed patients has been performed, demonstrating a prevalence of PTSD symptoms in 5/41 (12.2%) of participants (Qi et al., 2020). However, only non‐severe types of COVID‐19 cases were included, and the median time interval between hospitalization and PTSD assessment was less than one month. On the other hand, in addition to the traditional risk factors for PTSD such as age, sex, smoking, and alcohol drinking, factors associated with the severity of COVID‐19 (including peripheral blood indicators, hospitalization days, severe pneumonia, hypoalbuminemia, liver injury, and other severe complications) and patient metabolic characteristics (e.g., obesity) might be associated with risk of developing PTSD (Lee et al., 2018; Wang et al., 2020). Furthermore, information on personal and family history of diseases was available in this study. Thus, we conducted this prospective study with a relatively long‐term follow‐up to investigate the factors potentially involved in PTSD among confirmed COVID‐19 patients with various clinical types by including numerous predictors.

## 2 METHODS

### 2.1 Participants

From January 19 to April 30, 2020, a total of 144 participants were recruited from Tianjin Haihe Hospital, which is a designated hospital for COVID‐19 in Tianjin, China. This was a convenience sample of survivors. All participants provided written consent and all the protocol of this study was approved by the Institutional Review Board of Tianjin Haihe Hospital.

A SARS‐CoV‐2 virus‐specific real‐time reverse transcriptase polymerase chain reaction (RT–PCR) assay of respiratory specimens, including nasal and pharyngeal swabs is routinely used for diagnosis of COVID‐19 (Huang et al., 2020). We included all participants with laboratory‐confirmed SARS‐CoV‐2 infection.

### 2.2 Assessment of PTSD

PTSD symptoms were assessed using the impact of events scale‐revised (IES‐R) by telephone follow‐up, which was conducted by the same investigator around three months after laboratory‐confirmed SARS‐CoV‐2 infection. The shortest and longest timepoints of the follow‐up were 85 days and 93 days, respectively. Since PTSD symptoms usually begin within 3 months of the traumatic incident (Information MH. Post‐Traumatic Stress Disorder, 2021), we chose three months as the follow‐up timepoint. The IES‐R is a validated 22‐item self‐reporting questionnaire that measures the subjective distress caused by traumatic events. The 22‐item scale comprises three subscales: intrusion, avoidance, and hyperarousal, which correspond to the 3 PTSD symptom clusters. Participants were asked to indicate the degree with which they experienced each PTSD symptom in the past week on a 5‐point scale: 0 = “Not at all”, 1 = “A little bit”, 2 = “Moderately”, 3 = “Quite a bit”, 4 = “Extremely”. Scores ≥25 were considered to be of clinical concern (Rash et al., 2008). The IES‐R is the most widely used measure internationally for the psychological impact of trauma, and high reliability and validity were observed in the Chinese version of the IES‐R (Wu & Chan, 2003).

### 2.3 Laboratory measurements

Venous blood samples were routinely drawn from each patient in the fasting state at the earliest time points possible upon hospitalization. White blood cell count, lymphocyte count, hemoglobin, red blood cell count, fibrinogen, D‐dimer, C‐reactive protein (CRP), procalcitonin, albumin, alanine aminotransferase (ALT), aspartate aminotransferase (AST), alkaline phosphatase, creatine kinase, creatine kinase isoenzyme, lactate dehydrogenase, myoglobin, creatinine, urea nitrogen, as well as interleukin‐6, were measured under standard hospital assays run by the Department of Laboratory Medicine of the hospital.

### 2.4 CURB‐65 score

Data for the calculation of CURB‐65 were recorded at hospital admission. The CURB‐65 severity score (range 0–5 points) was calculated by attributing one point to each of the following clinical features present: confusion, urea > 7 mmol/L, respiratory rate ≥30/min, systolic blood pressure < 90 mmHg, and/or diastolic blood pressure 60 mmHg, and age 65 years (Lim et al., 2003).

### 2.5 Thin‐section CT imaging and scoring

Thin‐section computed tomography (CT) scans (Aquillion Prime 128, Canon Medical Systems, Otawara, Japan) were performed in all patients. Thin‐section CT was performed from the lung apices to the adrenal glands at full inspiration, and the process was later repeated at full expiration. The CT scanning parameters were: 64 × 0.5 mm collimation, 120 kV, automatic tube current modulation, 0.5 s gantry rotation time. Images were reconstructed with a hybrid iterative reconstruction algorithm (AIDR 3D) at mild settings, and contiguous inspiratory thin‐section CT images were obtained with a thickness of 1 mm at 0.8 mm intervals. CT scans were interpreted at window settings that were optimal for lung parenchyma (window level, −600 HU; window width, 1500 HU) and soft tissue (window level, 400 HU; window width, 40 HU). Patients were scanned while lying supine and during full inspiration and expiration. All CTs were scored by two experienced radiologists blinded to laboratory values and the medical history of the participants. Definitions of radiological terms like ground‐glass opacity, reticulation, honeycombing, parenchymal bands, consolidation, air trapping, and bronchiectasis were based on the recommendations of the Nomenclature Committee of the Fleischner Society (Hansell et al., 2008).

To quantify the extent of disease, a semi‐quantitative CT score was defined as the sum of lung involvement (0, no involvement; 1, < 5% involvement; 2, 5−25% involvement; 3, 26−50% involvement; 4, 51−75% involvement; and 5, > 75% involvement) of each lobe (Chang et al., 2005; Pan et al., 2020).

### 2.6 Measurement of other variables

Age, sex, alcohol drinking status (never or drinking), history of diseases (hypertension, diabetes mellitus, and heart disease), hospitalization days, and oxygenation index (OI) were obtained from a detailed questionnaire survey and electronic medical records. Data on smoking status were collected by a question: “Do you usually smoke?” with two response options: yes (current) or no (former/never). Height and body weight were measured with participants wearing light clothes and no shoes. Body mass index (BMI) was calculated as weight in kilograms divided by height in meters squared (kg/m^2^).

### 2.7 Complication definitions

Severe pneumonia was defined by the 2007 Infectious Disease Society of America/American Thoracic Society (IDSA/ATS) guidelines (Mandell et al., 2007). Two major criteria include the need for invasive mechanical ventilation and septic shock with the need for vasopressors. The minor criteria include respiratory rate ≥30 times per minute, PaO2/FiO2 ratio ≤250 (or need for noninvasive ventilation), multilobar infiltration, confusion/disorientation, uremia (BUN >20 mg/dL), leukopenia (<4000 cells/mm^3^ caused by infection), thrombocytopenia (<100,000 cells/mm^3^), hypothermia (<36°C), and hypotension requiring aggressive fluid resuscitation. The presence of at least one major criterion or three or more minor criteria was defined as severe pneumonia.

The normal range of serum albumin was 35–55 g/L. Based on previous research, hypoalbuminemia was defined as serum albumin level less than 35 g/L (Adogwa et al., 2014; Aldebeyan et al., 2017; Egbert et al., 2020). Acute liver injury was defined as jaundice with a total bilirubin level of ≥3 mg/dL combined with one of the following two abnormal levels of liver enzymes: (Worldometer, 2020) ≥5 times acute increase of the upper limit of the normal range in ALT; (Talevi et al., 2020) ≥2 times of the upper limit of the normal range in AST (Chen et al., 2020).

### 2.8 Statistical analysis

Statistical Analysis System 9.3 edition for Windows (SAS Institute Inc., Cary, NC) was used for data analysis. The descriptive data were expressed as counts (percentages) for categorical variables and medians (interquartile ranges) for continuous variables. Univariable logistic regression was used to analyze the association of PTSD and every potential independent variable, including age, sex, BMI, smoke status, alcohol‐drinking status, history of diseases (hypertension, diabetes mellitus, and heart disease), severe pneumonia, hypoalbuminemia, hepatic injury, other severe complications (acute kidney injury, respiratory failure, acute respiratory distress syndrome, arrhythmia, and cardiac insufficiency), CT score, and the number of involved pulmonary lobes, CURB‐65 score, hospitalization days, OI, white blood cell count, lymphocyte count, hemoglobin, red blood cell count, fibrinogen, D‐dimer, CRP, procalcitonin, albumin, ALT, AST, alkaline phosphatase, creatine kinase, creatine kinase isoenzyme, lactate dehydrogenase, myoglobin, creatinine, and urea nitrogen, separately. These variables were potential factors associated with PTSD (Lee et al., 2018; Wang et al., 2020). Stepwise logistic regression analysis with inclusion and exclusion criteria of *p *< .20 was performed to screen statistically significant the factors associated with PTSD (Chowdhury & Turin, 2020). No significant multicollinearity was found when the variance inflation factor was assessed. All tests were two‐tailed and statistical significance was set as *p *< .05.

## 3 RESULTS

Of the 144 participants with laboratory‐confirmed COVID‐19, the incidence of PTSD in all subjects was 16.0% (23/144). The incidence of PTSD was 9.72% (7/72) in males and 22.2% (16/72) in females, respectively. Baseline participant characteristics are displayed in Table 1.

**TABLE 1:** Characteristics of study participants

Characteristics	*n* = 144
PTSD, *n* (%)	23 (16.0)
Age (≥40y), *n* (%)	77 (53.5)
Males, *n* (%)	72 (50.0)
Obesity (BMI ≥24 kg/m^2^), *n* (%)	72 (50.0)
Smoker, *n* (%)	18 (12.5)
Drinker, *n* (%)	110 (76.4)
Medical history, *n* (%)	
Hypertension	22 (15.3)
Diabetes mellitus	10 (6.94)
Heart disease	4 (2.78)
Severe pneumonia	5 (3.47)
Complications, *n* (%)	
Hypoalbuminemia	12 (8.33)
Acute liver injury	38 (26.4)
Other severe complications	15 (10.4)
CRP ≥10 mg/l, *n* (%)	49 (34.0)
CT score ≥15, *n* (%)	23 (16.0)
Involved pulmonary lobes ≥3, *n* (%)	93 (64.6)
Hospitalization days (d)	14.0 (9.00, 20.5)
CURB‐65 score ≥1, *n* (%)	13 (9.0)
Oxygenation index	425.0 (322.0, 506.0)
White blood cell count (10^9^/L)	4.65 (3.69, 5.85)
Lymphocyte count (10^9^/L)	1.14 (0.85, 1.56)
Hemoglobin (g/L)	133.0 (126.0, 48.0)
Red blood cell count (10^12^/L)	4.28 (3.94, 4.70)
Fibrinogen (g/L)	2.90 (2.26, 3.71)
D‐dimer (μg/mL)	0.44 (0.25, 0.71)
CRP (mg/L)	6.04 (0.96, 29.12)
Procalcitonin (ng/mL)	0.04 (0.04, 0.04)
Albumin (g/L)	42.6 (39.3, 45.0)
Alanine aminotransferase (U/L)	33.5 (25.0, 47.0)
Aspartate aminotransferase (U/L)	29.0 (23.0, 38.0)
Alkaline phosphatase (U/L)	61.0 (51.0, 82.0)
Creatine kinase (U/L)	62.0 (41.0, 94.0)
Creatine kinase isoenzyme (ng/ml)	7.00 (5.00, 10.0)
Lactate dehydrogenase (U/L)	483.0 (412.0, 595.0)
Myoglobin (μg/L)	27.8 (19.4, 46.5)
Creatinine (μmol/L)	55.0 (45.0, 68.0)
Urea nitrogen (mmol/L)	4.00 (4.00, 5.00)
Interleukin‐6 (pg/mL)	4.10 (1.50, 15.0)

*Note*: Continuous variables were described as medians (interquartile ranges), and categorical variables were described as counts (percentages).

Abbreviations: BMI, body mass index; CRP, C‐reactive protein, CT, computed tomography; PTSD, post‐traumatic stress disorder.

The univariable and stepwise logistic regression analyses of possible risk factors and PTSD are shown in Table 2. In univariable logistic regression analysis, age above 40 years (OR [95% CI], 2.88 [1.11, 8.44]), female sex (OR [95% CI], 2.65 [1.05, 7.33]), current smoker (OR [95% CI], 4.40 [1.40, 12.9]), three or more involved pulmonary lobes (OR [95% CI], 4.40 [1.40, 19.3]), and a higher levels of urea nitrogen (OR [95% CI], 1.26 [1.04, 1.64]) were associated with a higher risk of PTSD, while a higher level of albumin (OR [95% CI], 0.89 [0.80, 0.97]) was associated with a lower risk of PTSD. In the stepwise logistic regression analysis, age above 40 years, female sex, current smoker, history of hypertension, three or more involved pulmonary lobes, and hemoglobin were identified as factors associated with PTSD, with ORs (95% CIs) of 4.42 (1.11, 20.2), 10.9 (2.04, 118.1), 60.09 (7.81, 232.55), 0.06 (0.00, 0.51), 4.70 (1.23, 19.9), 5.34 (1.09, 35.7), and 0.94 (0.87, 0.99), respectively.

**TABLE 2:** Univariable and stepwise logistic regression analyses of factors and PTSD in the study participants (*n* = 144)

Factors	No. of subjects	No. of PTSD	OR (95% CI)^a^	*p*‐Value^a^	OR (95% CI) ^b^	*p*‐Value^b^
Age				.04		.04
<40y	67	6	1.00 (reference)		1.00 (reference)	
≥40y	77	17	2.88 (1.11, 8.44)		4.42 (1.11, 20.2)	
Sex				.04		.02
Males	72	7	1.00 (reference)		1.00 (reference)	
Females	72	16	2.65 (1.05, 7.33)		10.9 (2.04, 118.1)	
BMI (kg/m^2^)				.50		.59
<24	72	10	1.00 (reference)		1.00 (reference)	
≥24	72	13	1.37 (0.56, 3.43)		0.70 (0.19, 2.54)	
Smoker				.01		<.001
No	126	16	1.00 (reference)		1.00 (reference)	
Yes	18	7	4.40 (1.40, 12.9)		60.09 (7.81, 232.55)	
Drinker				.45		–
No	34	4	1.00 (reference)		–	
Yes	110	19	1.57 (0.54, 5.72)		–	
History of hypertension				.76		.02
No	122	19	1.00 (reference)		1.00 (reference)	
Yes	22	4	1.21 (0.32, 3.67)		0.06 (0.00, 0.51)	
History of diabetes mellitus				.60		–
No	134	22	1.00 (reference)		–	
Yes	10	1	0.57 (0.03, 3.24)		–	
History of heart disease				.62		–
No	140	22	1.00 (reference)		–	
Yes	4	1	1.79 (0.09, 14.7)		–	
Severe pneumonia				.16		.11
No	139	21	1.00 (reference)		1.00 (reference)	
Yes	5	2	3.75 (0.47, 23.93)		7.97 (0.59, 114.29)	
Hypoalbuminemia				.38		–
No	132	20	1.00 (reference)		–	
Yes	12	3	1.87 (0.39, 6.90)		–	
Acute liver injury				.14		–
No	106	14	1.00 (reference)		–	
Yes	38	9	2.04 (0.78, 5.16)		–	
Other severe complications				.65		–
No	129	20	1.00 (reference)		–	
Yes	15	3	1.36 (0.29, 4.77)		–	
CRP (mg/l)				.57		–
<10	95	14	1.00 (reference)		–	
≥10	49	9	1.30 (0.50, 3.23)		–	
CT score				.16		–
<15	121	17	1.00 (reference)		–	
≥15	23	6	2.16 (0.70, 6.06)		–	
No. of involved pulmonary lobes				.02		.04
<3	51	3	1.00 (reference)		1.00 (reference)	
≥3	93	20	4.40 (1.40, 19.3)		5.34 (1.09, 35.7)	
Hospitalization days ≥15				.34		–
No	82	11	1.00 (reference)		–	
Yes	62	12	1.55 (0.63, 3.84)		–	
CURB‐65 score ≥2				.47		–
No	131	20	1.00 (reference)		–	
Yes	13	3	1.67 (0.35, 6.02)		–	
Oxygenation index	144	23	0.997 (0.99, 1.001)	.14	–	–
White blood cell count (10^9^/L)	144	23	1.19 (0.96, 1.46)	.10	–	–
Lymphocyte count (10^9^/L)	144	23	0.92 (0.37, 2.14)	.86	–	–
Hemoglobin (g/L)	144	23	0.98 (0.95, 1.006)	.13	0.94 (0.87, 0.99)	.03
Red blood cell count (10^12^/L)	144	23	0.38 (0.13, 1.002)	.07	–	–
Fibrinogen (g/L)	144	23	1.39 (0.96, 2.01)	.08	–	–
D‐dimer (μg/mL)	144	23	1.08 (0.48, 2.02)	.84	–	–
Procalcitonin (ng/mL)	144	23	1.69 (0.02, 41.3)	.75	–	–
Albumin (g/L)	144	23	0.89 (0.80, 0.97)	.01	–	–
Alanine aminotransferase (U/L)	144	23	0.99 (0.97, 1.01)	.50	–	–
Aspartate aminotransferase (U/L)	144	23	1.002 (0.98, 1.02)	.88	–	–
Alkaline phosphatase (U/L)	144	23	0.998 (0.99, 1.01)	.65	–	–
Creatine kinase (U/L)	144	23	1.001 (0.998, 1.002)	.53	–	–
Creatine kinase isoenzyme (ng/ml)	144	23	1.01 (0.99, 1.03)	.34	1.04 (0.99, 1.13)	.19
Lactate dehydrogenase (U/L)	144	23	1.001 (1.000, 1.003)	.11	–	–
Myoglobin (μg/L)	144	23	1.002 (0.999, 1.006)	.16	–	–
Creatinine (umol/L)	144	23	1.01 (0.99, 1.04)	.24	–	–
Urea nitrogen (mmol/L)	144	23	1.26 (1.04, 1.64)	.04	–	–
Interleukin‐6 (pg/mL)	144	23	1.01 (0.99, 1.02)	.55	–	–

Abbreviations: BMI, body mass index; CI, confidence interval; CRP, C‐reactive protein, CT, computed tomography; OR, odds ratio; PTSD, post‐traumatic stress disorder.

^a^ Univariable logistic regression analysis.

^b^ Stepwise logistic regression analysis.

## 4 DISCUSSION

To our knowledge, this is the first prospective study to examine factors associated with PTSD in patients with confirmed COVID‐19 infection. The results showed that older age, gender female, current smoking, and the number of involved pulmonary lobes (≥3), are risk factors for PTSD, while hypertension and high hemoglobin levels are inversely associated with the risk of PTSD.

In this study, 16.0% of COVID‐19 patients had PTSD 3 months after infection. This incidence rate is higher than that reported for 41 COVID‐19 patients at less than 1 month after symptom onset, where 12.2% had PTSD symptoms (Qi et al., 2020). A possible explanation might be that patients who were severely ill were excluded and PTSD was detected over the relatively short term in that study. However, the rate presented in our study was similar to that among nurses exposed to COVID‐19 in Hubei, China, which showed that the incidence of PTSD was 16.83% (Wang et al., 2020). By contrast, a previous study reported that PTSD prevalence was 2.7% in home‐quarantined college students 1 month after the outbreak of the COVID‐19 epidemic (Tang et al., 2020). This difference corresponding to previous studies indicated that the prevalence of PTSD is higher among persons who are directly exposed to a disaster (Neria et al., 2008).

In the present study, older age and gender female are the main risk factors associated with PTSD in confirmed COVID‐19 patients. In line with our results, a 4‐year follow‐up study indicated that survivors of SARS with PTSD were more likely to be older and female (Hong et al., 2009). In addition, a previous study conducted in confirmed COVID‐19 patients indicated that COVID‐19 patients with mental health problems were more likely to be female (Qi et al., 2020). Two additional studies also found that being female was a strong predictor of PTSD symptoms (Gonzalez‐Sanguino et al., 2020; Liu et al., 2020). Similarly, animal studies have demonstrated different mood changes in rats related to sex, with female rats showing stronger emotional changes than their male counterparts (Yang et al., 2019). Collectively, these findings highlight that old age and gender female are the main risk factors associated with PTSD in confirmed COVID‐19 patients.

To our knowledge, no studies have investigated how smoking influences PTSD. In the current prospective study, we observed that smoking was positively associated with PTSD in COVID‐19 patients. Previous studies also suggested that smoking (nicotine) could cause poor mental health through neurotransmitter pathways (Picciotto et al., 2002; Plurphanswat et al., 2017). Moreover, evidence has suggested that quitting smoking was associated with a decrease in depressive symptoms (Rodriguez‐Cano et al., 2016), which are strong risk factors for PTSD (Momma et al., 2014). Thus, the integration of stop smoking services into the PTSD care pathway would be a good way to improve mental health.

Multivariable logistic regression analysis indicated that the COVID‐19 patients with ≥3 involved pulmonary lobes were more likely to have PTSD. Previous studies have suggested that COVID‐19 patients with ≥3 involved pulmonary lobes had more severe illness (Dudoignon et al., 2020). This might be explained by the fact that a high incidence of PTSD was associated with the severity of exposure to disaster (Lee et al., 2018).

In our study, we found that a high hemoglobin level may act as a protective factor for PTSD in COVID‐19 patients. This aligns with previous studies which documented that low hemoglobin level is a risk factor for postpartum PTSD (Sentilhes et al., 2017). These works also suggested that low baseline hemoglobin level strongly predicts incident depression (Trevisan et al., 2016). Taken together, this information suggests that anemia may contribute to PTSD in COVID‐19 patients. Indeed, the current study also showed anemia was a risk factor for PTSD, with adjusted OR = 1.59 (95% CI: 0.48–4.59). Our findings suggest that discharged COVID‐19 patients with high hemoglobin level had a low possibility of developing PTSD.

Interestingly, our study showed that baseline hypertension was inversely associated with the risk of PTSD. In contrast, a previous study demonstrated that a history of chronic illness was significantly associated with higher IES‐R scores among the general population in China (Wang et al., 2020). Furthermore, an inverse association between hypertension and depression has been previously reported (Briggs et al., 2016). Nevertheless, the inverse association between hypertension and PTSD should be interpreted with caution as the proportion of COVID‐19 patients with hypertension was small, resulting in low statistical power for this finding. Future research should investigate the role of hypertension in the development of PTSD using higher powered prospective studies.

To the best of our knowledge, there is no prospective study to identify the risk factors that develop PTSD in confirmed COVID‐19 patients. In this study, we collected many variables at baseline, such as smoking status, drinking status, medical history, complications, clinical parameters, and blood parameters, which have allowed us to prospectively investigate the factors associated with PTSD in COVID‐19 patients. Nevertheless, the present study has some limitations. First, PTSD was assessed by telephone follow‐up. However, the follow‐up was performed by a trained psychiatrist using a standardized interview technique. Second, although this is a prospective study, the baseline PTSD was not assessed. Third, we did not collect data on dietary intake, physical activity, and the history of psychiatry (depression, anxiety, schizophrenia, etc.), which might have affected the incidence of PTSD (Hall et al., 2015). In addition, either COVID‐related or non‐COVID‐related interventions such as clinical and/or follow‐up social support were not assessed in the study, so the residual confounding existed. Therefore, future research should be conducted to assess the effect of nutrition and exercise on PTSD. Fourth, our study had a relatively small size (144 patients) and a short follow‐up time. Therefore, future large‐scale prospective study with longer follow‐up time is needed. Finally, our study sample was from a hospital in Tianjin, China, potentially limiting generalizability to hospital settings elsewhere.

In conclusion, the present study showed that the incidence of PTSD was not rare among patients with confirmed COVID‐19 infection. We also found that older age, female sex, current smoking, and ≥3 involved pulmonary lobes were associated with increased risk of PTSD in COVID‐19 patients, whereas hypertension and high hemoglobin level were inversely associated with risk of PTSD in COVID‐19 patients.

## CONFLICT OF INTEREST

The authors declare no conflict of interest.

### PEER REVIEW

The peer review history for this article is available at https://publons.com/publon/10.1002/brb3.2492

## Data Availability

Data are available from the corresponding author (KN: nkj0809@gmail.com) upon reasonable request.

## References

[brb32492-bib-0001] Worldometer, (2020). https://www.worldometers.info/coronavirus/

[brb32492-bib-0002] Talevi, D. , Socci, V. , Carai, M. , Carnaghi, G. , Faleri, S. , Trebbi, E. , di Bernardo, A. , Capelli, F. , & Pacitti, F. (2020). Mental health outcomes of the CoViD‐19 pandemic. Rivista di Psichiatria, 55(3), 137–144. 32489190 10.1708/3382.33569

[brb32492-bib-0003] Vindegaard, N. , & Benros, M. E. (2020). COVID‐19 pandemic and mental health consequences: Systematic review of the current evidence. Brain, Behavior, and Immunity, 89, 531–542. 10.1016/j.bbi.2020.05.048 PMC7260522 32485289

[brb32492-bib-0004] Pfefferbaum, B. , & North, C. S. (2020). Mental health and the Covid‐19 pandemic. The New England Journal of Medicine, 383, 510–512. 32283003 10.1056/NEJMp2008017

[brb32492-bib-0005] Bo, H. X. , Li, W. , Yang, Y. , Wang, Y. , Zhang, Q. , Cheung, T. , Wu, X. , & Xiang, Y.‐T. (2020). Posttraumatic stress symptoms and attitude toward crisis mental health services among clinically stable patients with COVID‐19 in China. Psychological Medicine, 51(6),1052–1053. 32216863 10.1017/S0033291720000999 PMC7200846

[brb32492-bib-0006] Tsai, J. , Harpaz‐Rotem, I. , Pietrzak, R. H. , & Southwick, S. M. (2012). The role of coping, resilience, and social support in mediating the relation between PTSD and social functioning in veterans returning from Iraq and Afghanistan. Psychiatry, 75(2), 135‐49. 22642433 10.1521/psyc.2012.75.2.135

[brb32492-bib-0007] Galea, S. , Nandi, A. , & Vlahov, D. (2005). The epidemiology of post‐traumatic stress disorder after disasters. Epidemiologic Reviews, 27(1), 78–91. 15958429 10.1093/epirev/mxi003

[brb32492-bib-0008] Wu, K. , & Wei, X. (2020). Analysis of psychological and sleep status and exercise rehabilitation of front‐line clinical staff in the fight against covid‐19 in china. Medical Science Monitor Basic Research, 26, e924085. 32389999 10.12659/MSMBR.924085 PMC7241216

[brb32492-bib-0009] Wang, Y. X. , Guo, H. T. , Du, X. W. , Song, W. , Lu, C. , & Hao, W. N. (2020). Factors associated with post‐traumatic stress disorder of nurses exposed to corona virus disease 2019 in China. Medicine, 99(26), e20965. 32590808 10.1097/MD.0000000000020965 PMC7328992

[brb32492-bib-0010] Wang, C. , Pan, R. , Wan, X. , Tan, Y. , Xu, L. , McIntyre, R. S. , Choo, F. N. , Tran, B. , Ho, R. , Sharma, V. K. , & Ho, C. (2020). A longitudinal study on the mental health of general population during the COVID‐19 epidemic in China. Brain, Behavior, and Immunity, 87, 40–48. 10.1016/j.bbi.2020.04.028 PMC7153528 32298802

[brb32492-bib-0011] Tang, W. , Hu, T. , Hu, B. , Jin, C. , Wang, G. , Xie, C. , Chen, S. , & Xu, J. (2020). Prevalence and correlates of PTSD and depressive symptoms one month after the outbreak of the COVID‐19 epidemic in a sample of home‐quarantined Chinese university students. Journal of Affective Disorders, 274, 1–7. 32405111 10.1016/j.jad.2020.05.009 PMC7217769

[brb32492-bib-0012] Qi, R. , Chen, W. , Liu, S. , Thompson, P. M. , Zhang, L. J. , Xia, F. , Cheng, F. , Hong, A. , Surento, W. , Luo, S. , Sun, Z. Y. , Zhou, C. S. , Li, L. , Jiang, X. , & Lu, G. M. (2020). Psychological morbidities and fatigue in patients with confirmed COVID‐19 during disease outbreak: Prevalence and associated biopsychosocial risk factors. medRxiv : the preprint server for health sciences.

[brb32492-bib-0013] Lee, S. H. , Kim, E. J. , Noh, J. W. , & Chae, J. H. (2018). Factors associated with post‐traumatic stress symptoms in students who survived 20 months after the sewol ferry disaster in Korea. Journal of Korean Medical Science, 33(11), e90. 29495137 10.3346/jkms.2018.33.e90 PMC5835586

[brb32492-bib-0014] Huang, C. , Wang, Y. , Li, X. , Ren, L. , Zhao, J. , Hu, Y. , Zhang, L.i , Fan, G. , Xu, J. , Gu, X. , Cheng, Z. , Yu, T. , Xia, J. , Wei, Y. , Wu, W. , Xie, X. , Yin, W. , Li, H. , Liu, M. , …, & Cao, B. (2020). Clinical features of patients infected with 2019 novel coronavirus in Wuhan, China. The Lancet, 395(10223), 497–506. 10.1016/S0140-6736(20)30183-5 PMC7159299 31986264

[brb32492-bib-0015] Information MH. Post‐Traumatic Stress Disorder . (2021). https://www.nimh.nih.gov/health/publications/post‐traumatic‐stress‐disorder‐ptsd

[brb32492-bib-0016] Rash, C. J. , Coffey, S. F. , Baschnagel, J. S. , Drobes, D. J. , & Saladin, M. E. (2008). Psychometric properties of the IES‐R in traumatized substance dependent individuals with and without PTSD. Addictive Behaviors, 33(8), 1039–1047. 18501524 10.1016/j.addbeh.2008.04.006 PMC2494706

[brb32492-bib-0017] Wu, K. K. , & Chan, K. S. (2003). The development of the Chinese version of impact of event scale–revised (CIES‐R). Social Psychiatry and Psychiatric Epidemiology, 38(2), 94–98. 12563552 10.1007/s00127-003-0611-x

[brb32492-bib-0018] Lim, W. S. , van der Eerden, M. M. , Laing, R. , Boersma, W. G. , Karalus, N. , Town, G. I. , Lewis, S. A. , & Macfarlane, J. T. (2003). Defining community acquired pneumonia severity on presentation to hospital: An international derivation and validation study. Thorax, 58(5), 377–382. 12728155 10.1136/thorax.58.5.377 PMC1746657

[brb32492-bib-0019] Hansell, D. M. , Bankier, A. A. , MacMahon, H. , McLoud, T. C. , Muller, N. L. , & Remy, J. (2008). Fleischner Society: Glossary of terms for thoracic imaging. Radiology, 246(3), 697–722. 18195376 10.1148/radiol.2462070712

[brb32492-bib-0020] Pan, F. , Ye, T. , Sun, P. , Gui, S. , Liang, B. , Li, L. , Zheng, D. , Wang, J. , Hesketh, R. L. , Yang, L. , & Zheng, C. (2020). Time course of lung changes at chest ct during recovery from coronavirus disease 2019 (COVID‐19). Radiology, 295(3), 715–721. 32053470 10.1148/radiol.2020200370 PMC7233367

[brb32492-bib-0021] Chang, Y. C. , Yu, C. J. , Chang, S. C. , Galvin, J. R. , Liu, H. M. , Hsiao, C. H. , Kuo, P.‐H. , Chen, K.‐Y. , Franks, T. J. , Huang, K.‐M. , & Yang, P.‐C. (2005). Pulmonary sequelae in convalescent patients after severe acute respiratory syndrome: Evaluation with thin‐section CT. Radiology, 236(3), 1067–1075. 16055695 10.1148/radiol.2363040958

[brb32492-bib-0022] Mandell, L. A. , Wunderink, R. G. , Anzueto, A. , Bartlett, J. G. , Campbell, G. D. , Dean, N. C. , Dowell, S. F. , File, T. M. , Musher, D. M. , Niederman, M. S. , Torres, A. , & Whitney, C. G. (2007). Infectious Diseases Society of America/American Thoracic Society consensus guidelines on the management of community‐acquired pneumonia in adults. Clinical Infectious Diseases, 44(Suppl 2), S27–72. 17278083 10.1086/511159 PMC7107997

[brb32492-bib-0023] Egbert, R. C. , Bouck, T. T. , Gupte, N. N. , Pena, M. M. , Dang, K. H. , Ornell, S. S. , & Zelle, B. A. (2020). Hypoalbuminemia and obesity in orthopaedic trauma patients: Body mass index a significant predictor of surgical site complications. Scientific Reports, 10(1), 1953. 32029855 10.1038/s41598-020-58987-4 PMC7004978

[brb32492-bib-0024] Adogwa, O. , Martin, J. R. , Huang, K. , Verla, T. , Fatemi, P. , Thompson, P. , Cheng, J. , Kuchibhatla, M. , Lad, S. P. , Bagley, C. A. , & Gottfried, O. N. (2014). Preoperative serum albumin level as a predictor of postoperative complication after spine fusion. Spine (Phila Pa 1976), 39(18), 1513–1519. 24875960 10.1097/BRS.0000000000000450

[brb32492-bib-0025] Aldebeyan, S. , Nooh, A. , Aoude, A. , Weber, M. H. , & Harvey, E. J. (2017). Hypoalbuminaemia‐a marker of malnutrition and predictor of postoperative complications and mortality after hip fractures. Injury, 48(2), 436–440. 28040258 10.1016/j.injury.2016.12.016

[brb32492-bib-0026] Chen, G. , Wu, D. , Guo, W. , Cao, Y. , Huang, D. , Wang, H. , Wang, T. , Zhang, X. , Chen, H. , Yu, H. , Zhang, X. , Zhang, M. , Wu, S. , Song, J. , Chen, T. , Han, M. , Li, S. , Luo, X. , Zhao, J. , & Ning, Q. (2020). Clinical and immunological features of severe and moderate coronavirus disease 2019. Journal of Clinical Investigation, 130(5), 2620–2629. 10.1172/JCI137244 PMC7190990 32217835

[brb32492-bib-0027] Chowdhury, M. Z. I. , & Turin, T. C. (2020). Variable selection strategies and its importance in clinical prediction modelling. Family Medicine and Community Health, 8(1), e000262. 32148735 10.1136/fmch-2019-000262 PMC7032893

[brb32492-bib-0028] Neria, Y. , Nandi, A. , & Galea, S. (2008). Post‐traumatic stress disorder following disasters: A systematic review. Psychological Medicine, 38(4), 467–480. 17803838 10.1017/S0033291707001353 PMC4877688

[brb32492-bib-0029] Hong, X. , Currier, G. W. , Zhao, X. , Jiang, Y. , Zhou, W. , & Wei, J. (2009). Posttraumatic stress disorder in convalescent severe acute respiratory syndrome patients: A 4‐year follow‐up study. General Hospital Psychiatry, 31(6), 546–554. 19892213 10.1016/j.genhosppsych.2009.06.008 PMC7112559

[brb32492-bib-0030] Liu, N. , Zhang, F. , Wei, C. , Jia, Y. , Shang, Z. , Sun, L. , Wu, L. , Sun, Z. , Zhou, Y. , Wang, Y. , & Liu, W. (2020). Prevalence and predictors of PTSS during COVID‐19 outbreak in China hardest‐hit areas: Gender differences matter. Psychiatry Research, 287, 112921. 32240896 10.1016/j.psychres.2020.112921 PMC7102622

[brb32492-bib-0031] Gonzalez‐Sanguino, C. , Ausin, B. , Castellanos, M. A. , Saiz, J. , Lopez‐Gomez, A. , Ugidos, C. , & Muñoz, M. (2020). Mental health consequences during the initial stage of the 2020 Coronavirus pandemic (COVID‐19) in Spain. Brain, Behavior, and Immunity, 87, 172–176. 10.1016/j.bbi.2020.05.040 PMC7219372 32405150

[brb32492-bib-0032] Yang, R. , Sun, H. , Wu, Y. , Lu, G. , Wang, Y. , Li, Q. , Zhou, J. , Sun, H. , & Sun, L. (2019). Long‐lasting sex‐specific effects based on emotion‐ and cognition‐related behavioral assessment of adult rats after post‐traumatic stress disorder from different lengths of maternal separation. Frontiers in Psychiatry, 10, 289. 31231246 10.3389/fpsyt.2019.00289 PMC6558979

[brb32492-bib-0033] Plurphanswat, N. , Kaestner, R. , & Rodu, B. (2017). The effect of smoking on mental health. American Journal of Health Behavior, 41(4), 471–483. 28601107 10.5993/AJHB.41.4.12

[brb32492-bib-0034] Picciotto, M. R. , Brunzell, D. H. , & Caldarone, B. J. (2002). Effect of nicotine and nicotinic receptors on anxiety and depression. Neuroreport, 13(9), 1097–1106. 12151749 10.1097/00001756-200207020-00006

[brb32492-bib-0035] Rodriguez‐Cano, R. , Lopez‐Duran, A. , del Rio, E. F. , Martinez‐Vispo, C. , Martinez, U. , & Becona, E. (2016). Smoking cessation and depressive symptoms at 1‐, 3‐, 6‐, and 12‐months follow‐up. Journal of Affective Disorders, 191, 94–99. 26655118 10.1016/j.jad.2015.11.042

[brb32492-bib-0036] Momma, H. , Niu, K. , Kobayashi, Y. , Huang, C. , Otomo, A. , Chujo, M. , Tadaura, H. , & Nagatomi, R. (2014). Leg extension power is a pre‐disaster modifiable risk factor for post‐traumatic stress disorder among survivors of the Great East Japan Earthquake: A retrospective cohort study. Plos One, 9(4), e96131. 24760054 10.1371/journal.pone.0096131 PMC3997555

[brb32492-bib-0037] Dudoignon, E. , Camelena, F. , Deniau, B. , Habay, A. , Coutrot, M. , Ressaire, Q. , Plaud, B. , Berçot, B. , & Dépret, F. (2021). Bacterial pneumonia in COVID‐19 critically ill patients: A case series. Clinical Infectious Diseases: An Official Publication of the Infectious Diseases Society of America, *72*(5), 905–906. 10.1093/cid/ciaa762 PMC7337703 32544219

[brb32492-bib-0038] Sentilhes, L. , Maillard, F. , Brun, S. , Madar, H. , Merlot, B. , Goffinet, F. , & Deneux‐Tharaux, C. (2017). Risk factors for chronic post‐traumatic stress disorder development one year after vaginal delivery: A prospective, observational study. Scientific Reports, 7(1), 8724. 28821837 10.1038/s41598-017-09314-x PMC5562814

[brb32492-bib-0039] Trevisan, C. , Veronese, N. , Bolzetta, F. , De Rui, M. , Correll, C. U. , Zambon, S. , Musacchio, E. , Sartori, L. , Perissinotto, E. , Crepaldi, G. , Solmi, M. , Manzato, E. , & Sergi, G. (2016). Low hemoglobin levels and risk of developing depression in the elderly: Results from the prospective pro.v.a. study. The Journal of Clinical Psychiatry, 77(12), e1549–e1556. 27835718 10.4088/JCP.15m10270

[brb32492-bib-0040] Briggs, R. , Kenny, R. A. , & Kennelly, S. P. (2016). Systematic review: The association between late life depression and hypotension. Journal of the American Medical Directors Association, 17(12), 1076–1088. 27523406 10.1016/j.jamda.2016.06.027

[brb32492-bib-0041] Hall, K. S. , Hoerster, K. D. , & Yancy, W. S. Jr . (2015). Post‐traumatic stress disorder, physical activity, and eating behaviors. Epidemiologic Reviews, 37, 103–115. 25595169 10.1093/epirev/mxu011

